# Artificial Dentures

**Published:** 1860-01

**Authors:** J. Allen


(To be continued.)

### ARTICLE II.

### *Artificial Dentures.* BY DR. J. ALLEN.

ALTHOUGH more than two thousand years have passed since artificial teeth were first worn by man, and although a variety of methods have been introduced within that period of time, still there are errors, (whatever be the mode) to which the writer deems it proper to refer, in a series of articles upon this subject.

In taking a retrospective view of the past, we find that since the establishment of dental colleges, dental journals, and dental associations, in America, greater advances have been made in dental science, than during the previous twelve hundred years. Hence the universal concession that American dentists stand the highest in their profession.

Not that all these advances, or improvements, have emanated directly from these sources, but they have been instrumental in diffusing light to the profession, and light reveals principles which beget thought. A principle may be discovered and proclaimed by one person, which is often carried out and rendered practical by another, and again, modified and clothed with a different garb by a third. These means of diffusing knowledge, cause ideas to germinate, bud, bloom, and mature into substantial realities. Although much has been done in the construction of artificial dentures by means of the various devices which have been conceived and brought into use for this purpose, yet there are many errors, into which the dentist almost imperceptibly falls, either from want of sufficient perception to see the end in the beginning of his operations, or from a degree of carelessness in his manipulations, either of which may prove fatal to a practical result. To some of these errors we will briefly refer and endeavor to point out the remedies. There are those who attach too *much* importance to some favorite method, and too *little*, to its execution. They seem to think that this or that particular *mode* will almost do itself, and that all the genius, skill and judgment, that were necessary to be expended upon it, had been embodied in developing the *mode*, and the operator has only to employ it in order to produce a perfect result. This is an error and should be the first corrected. There is no one method, that has yet been discovered, so perfect as to render the exercise of taste, skill, and judgment, unnecersary on the part of the operator. There are no mouths so near alike as to admit, of duplicating artificial dentures, and have them properly adapted to different persons. Almost any other manufactured article can be multiplied ad infinitum, but artificial teeth must be varied to suit the requirements of each individual case. Hence the importance of the combined skill of the mechanist, the artist and the dentist. If the operator is deficient in the first of these requirements, there will be a want of utility in his operations. This may arise from various causes; for example, suppose the teeth be placed too far out upon the base which would bring the pressure (when the teeth are closed) outside of the alveolar ridge. This is a very common error, and often defeats a good practical result.

The skillful architect builds his superstructure perpendicularly over his foundation, lest the weight or pressure should overhang the base, and cause it to give way.

The same principle should be observed in the construction of a set of artificial teeth. They should be placed as nearly as possible, perpendicularly over the alveolar ridge, with the pressure resting upon the inner, rather than the outer cusps of the molars and bicuspids when the teeth are closed. This will secure not only strength to the denture, but also prevent the plate from becoming dislodged from one side, when chewing upon the opposite. Again a denture may be defective from an imperfectly fitting plate. If a zinc cast be used for swaging, the shrinkage of the metal will often prove sufficient to prevent a perfect fit to the mouth. To remedy this, another die should be made of fusible metal from the same plaster model from which the zinc one was formed. With this fusible die, the plate should again be struck, and if done with care, the plate will then be formed to fit, provided the impression was good. The fusible metal shrinks less than the zinc and is the best to be used for the last striking of the plate. The operator may also fail in the adaptation and arrangement of the teeth. If they are too light, they present a cold ghastly appearance which leads to detection.

They should possess a depth of tone, and shade, that will harmonize with the tinge of the lips, and complexion of the person for whom they are intended. If too dark, they exhibit a dirty and unhealthy appearance. If the teeth are set very true and even, they will appear stiff, and mechanical, and serve as a walking advertisement for the dentist who inserted them. They should be gracefully irregular, so that each tooth may display its natural individuality.

The expression of the teeth gives character to the physiognomy of a person which may be varied by longer or shorter, larger or smaller, prominent or receding teeth, either of which may produce harmony with the other features of one person, and distortion in the mouth of another. Careful discrimination and good judgment must decide as to the style and character of teeth that will give the best expression under the circumstances that govern the case. The teeth should be long enough to sustain a due proportion in the length of the face, and also display an agreeable and natural expression, and short enough to allow the lips to close over them without restraint, or undue muscular effort.

Another very common defect in the construction of artificial dentures, is that of *not* restoring the natural form of the face in cases where it has become sunken.

Some attempt to accomplish this by placing the teeth far out upon the plate, and thus sacrifice utility for appearance, which they fail to attain by this means, for it requires something more. When the muscles of the face have become sunken, they should be brought out to their original fulness, by means of attachments to the denture of such form and size, as to restore a symmetrical contour to the face, these attachments may be made of substances which can be united to the base plate and teeth in such manner as to become permanent fixtures to the denture ; the writer forms them exclusively with the materials used for his continuous gum work ; they are built out, carved, and baked upon the denture, of such form and size as may be indicated by wax, previously formed in proper shape upon the piece, when in the mouth of the patient. The skill of the artist is here brought into requisition, for he displays his talents upon the living features of the face ; he should know the origin, and insertion, of every muscle of which it is formed, and what ones he is to raise, and where to apply his attachments, or he may produce distortion instead of restoration, by allowing them to underlay other muscles than those intended to be raised.

If skill and judgment have presided over all parts of the operation, the result will be highly pleasing and of practical utility.

